# Cochlear implantation in a patient with congenital microtia, cochlear hypoplasia, venous anomalies of the temporal bone and laryngomalacia: Challenges and surgical considerations

**DOI:** 10.1097/MD.0000000000033000

**Published:** 2023-02-17

**Authors:** Xue Gao, Juan Zhao, Guan-Hua Li, Xi Wang, Wei Wang, Xing Liu, Min Liu, Meng-Meng Guo, Zhen-Dong Wang, Ya-Yan Lu, Jia Li, Yong Feng, Kun Yang, Jin-Cao Xu, Pu Dai

**Affiliations:** a College of Otolaryngology Head and Neck Surgery, Chinese PLA General Hospital, Chinese PLA Medical School, Beijing, China; b National Clinical Research Center for Otolaryngologic Diseases, State Key Lab of Hearing Science, Ministry of Education, China, Beijing Key Lab of Hearing Impairment Prevention and Treatment, Beijing, China; c Department of Otolaryngology, PLA Rocket Force Characteristic Medical Center, Beijing, P. R. China; d Department of Anesthesiology, PLA Rocket Force Characteristic Medical Center, Beijing, P. R. China; e Postgraduate Training Base of Jinzhou Medical University (The PLA Rocket Force Characteristic Medical Center), Beijing, P. R. China.

**Keywords:** cochlear implantation, congenital laryngomalacia, inner ear malformation, microtia, venous anomalies

## Abstract

Congenital hearing loss is often caused by an inner ear malformation, in such cases, the presence of other anomalies, such as microtia, and venous anomalies of the temporal bone and laryngomalacia makes it challenging to perform cochlear implantation surgery.

**Diagnoses:**

This study reports the case of a 28-month-old girl with congenital profound hearing loss, laryngomalacia, and malformed inner ear, who received cochlear implantation surgery. The bony structure, vessels and nerves were first assessed through magnetic resonance imaging and computed tomography before exploring the genetic basis of the condition using trio-based whole exome sequencing. Perioperative evaluation and management of the airway was then performed by experienced anesthesiologist, with the surgical challenges as well as problems encountered fully evaluated.

**Interventions:**

Cochlear implantation was eventually performed using a trans-mastoid approach under uneventful general anesthesia.

**Outcomes:**

Due to the small size of the cochlea, a short electrode FLEX24 was inserted through the cochleostomy.

**Lessons:**

Considering the high risk of facial nerve injury and limited access to the cochlea when patients present significant bony and venous anomalies, cochlear implantation in such patients require careful preoperative evaluation and thoughtful planning. In these cases, airway assessment, magnetic resonance venography, magnetic resonance arteriography, and magnetic resonance imaging and computed tomography can be useful to minimize the risks. Intraoperative facial nerve monitoring is also recommended to assist in the safe location of facial nerve.

## 1. Introduction

Being a well-established form of treatment, cochlear implantation (CI) is often performed in patients with bilateral severe to profound sensorineural hearing loss. Yet, while congenital hearing loss is commonly caused by inner ear malformation,^[1]^ prior to 2013, CI was contraindicated in patients presenting such malformations. However, this treatment was subsequently shown to be effective in most cases of inner ear malformation.^[2,3]^ This study presents the case of a 28-month-old with multiple anomalies who underwent CI before discussing the main surgical implications of the intervention.

## 2. Case presentation

**Figure 1.**
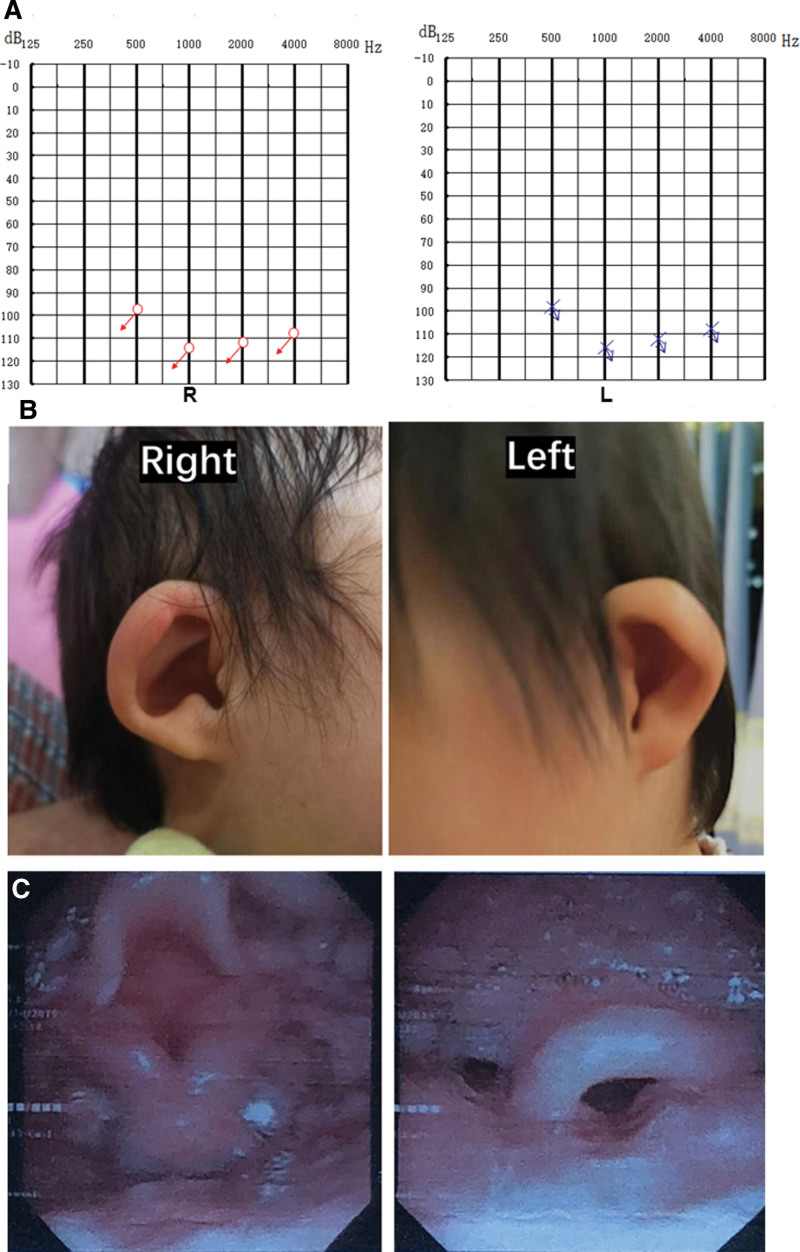
Physical examination of the patient. A. The auditory steady state response (ASSR) of the patient shows bilateral profound sensorineural hearing loss. All frequencies elicited no response. B. Both ears are smaller in size and are classified as grade II microtia. C. Flexible transnasal fiberoptic laryngoscopy shows that the epiglottis was slightly curled, when inhaling, the bilateral flap covered most of the glottis, epiglottis collapse, and the glottis was unclear.

**Figure 2.**
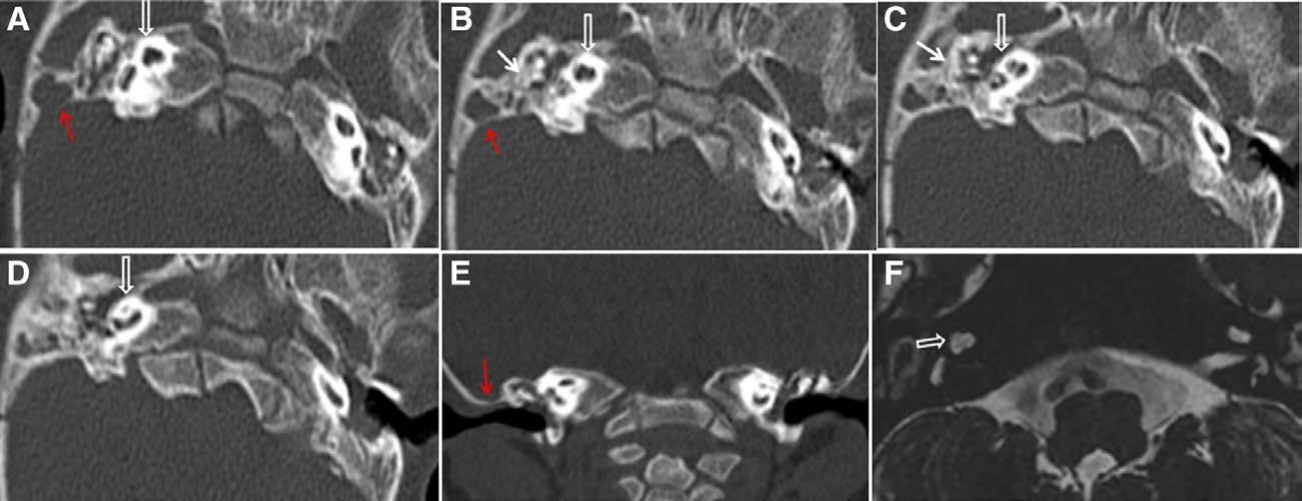
Preoperative radiologic images of the patient (CT and MRI). A-D. Axial computed tomography demonstrated bilateral low-lying tegmen (red arrows), poor pneumatization of the middle ear space (white arrow), absent lateral semicircular canals, cochlear hypoplasia (white open arrow). E. Coronal computed tomography demonstrated low-lying tegmen (red arrow). F. MRI revealed cochlear hypoplasia on the right side (white open arrow). CT= computed tomography, MRI = magnetic resonance imaging.

A 28-month-old girl with congenital profound hearing loss was comprehensively evaluated for CI. Physical examination revealed that the ears were smaller in size, but along with normal external ear canals, the key features of a normal ear were also present. Thus, the condition was classified as a grade II microtia (Fig. 1B).^[4]^ At the age of 6 months, the patient was diagnosed with profound sensorineural hearing loss in both ears (Fig. 1A). The patient experienced noisy breathing as well as respiratory distress since birth and a diagnosis of laryngomalacia was confirmed. Laryngoscopy revealed that the epiglottis was slightly curled, when inhaling, the flap covered most of the glottis, and thus making the glottis unclear (Fig. 1C). Preoperative magnetic resonance imaging (MRI) and computed tomography (CT) showed poor bilateral pneumatization of the middle ear space with neither mastoid antrum nor air cells. Absent lateral semicircular canals, cochlear hypoplasia, and narrow internal auditory canals bilaterally were found during preoperative radiological images. An aberrant low-lying tegmen was found to penetrate the temporal bone from the middle fossa and cover the surface of undeveloped antrum. However, the preoperative use of noncontrast MRI and CT did not show venous anomalies (Fig. 2). Similarly, based on whole exome sequencing of the trios, no pathologic or likely pathogenic variants were identified.

## 3. Operative details

**Figure 3.**
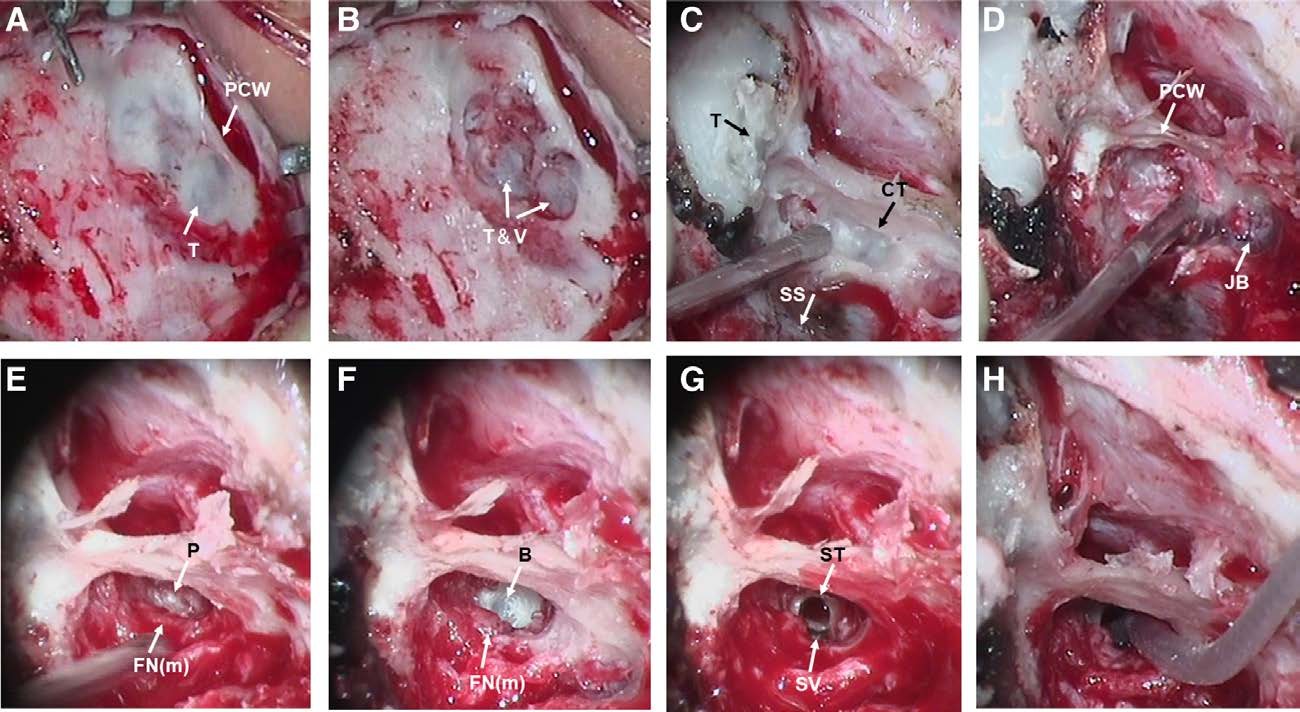
CI surgery. A. Drilling out the superficial bone of mastoid, an aberrant low-lying tegmen was seen penetrating the temporal bone from the middle fossa and cover the surface of undeveloped antrum. B. The vein sinus run on the surface of the tegmen and obstructed the standard approach to the cochleostomy middle fossa. C. Bipolar cautery was used for retracting the vein sinus by bipolar cautery and covered by bone wax. Using sigmoid sinus as landmarks, by removing the overlying bone with a fine diamond drill gradually in the deep part of the mastoid, the mastoid segment of the facial nerve still can not be identified. D. By removing the overlying bone in the deep part of the mastoid and expose the sigmoid sinus and jugular bulb, the mastoid segment of the facial nerve still can not be identified. E. mastoid segment of the facial nerve and promontory. F. drill the bone over promontory (basal turn). G. a cochleostomy was made on the promontory. H. The electrode was fully inserted through the basal turn cochleostomy. B = basal turn of cochlea, CI = cochlear implantation, CT= computed tomography , FN(m) = mastoid segment of the facial nerve, JB = jugular bulb, P = promontory, PCW = posterior canal wall, SS = sigmoid sinus, ST = Scala tympani, SV = Scala vestibuli, T = tegmen, V = vessel.

**Figure 4.**
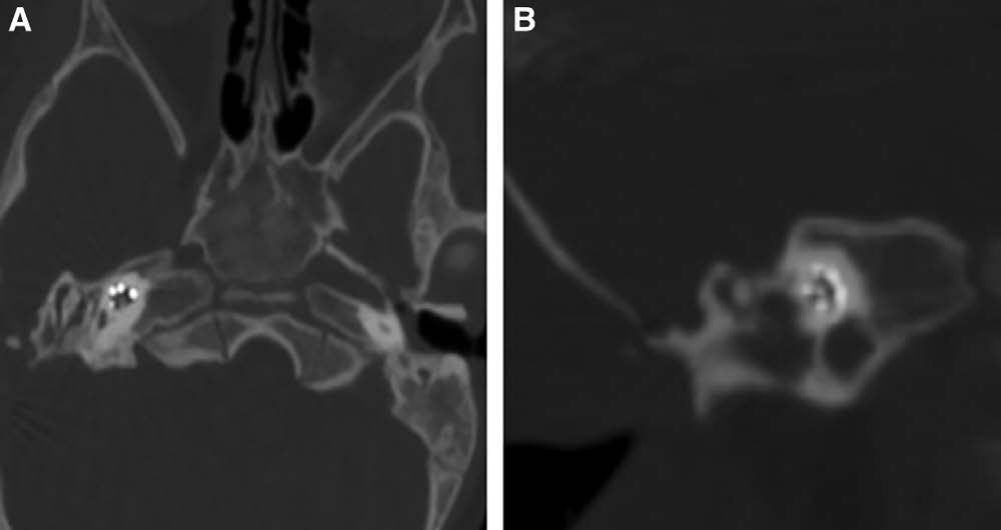
Postoperative CT of the patient. Axial (A) and coronal CT (B) showed all the electrodes were inserted into the cochlea (right side) and there was no folded or dislocation of the electrodes. CT = computed tomography.

CI was performed in the right ear but the standard procedure for cochleostomy was obstructed by the aberrant low-lying tegmen and vein sinus (Fig. 3A and B). Bipolar cautery was used for retracting the vein sinus into the cavity where it was covered with bone wax to protect the vein from bleeding (Fig. 3C). The antrum was not developed, the mastoid was completely sclerotic and the most important landmarks for identifying the mastoid segment of the facial nerve, including the incus and the lateral semicircular canal were absent or obstructed by tegmen. By removing the overlying bone and exposing the jugular bulb and sigmoid sinus, the mastoid segment of the facial nerve could still not be identified (Fig. 3D). Thus, the external auditory canal was used as access to identify the facial nerve. Using stapes, oval window as landmark, the tympanic segment of the facial nerve was located. The posterior ear canal wall was thinned to a “paper thin” thickness. The pyramid and mastoid segment of the facial nerve was thick and displaced more anteromedially than that of the normal ears (Fig. 3E). Hypoplasia of the round window was identified, and a diamond drill was used to for a cochleostomy in the promontory area (Fig. 3F and G). The electrode of Concerto F24 (MED-EL Corp, Austria) was fully inserted in the Scala tympani through the cochleostomy (shown in Fig. 3H). Clear neural response telemetry reactions were obtained on 1 electrode. Following the surgery, neither leakage of cerebrospinal fluid nor facial paralysis was observed in the patient. In addition, no intraoperative or postoperative complications occurred, with the inserted electrodes shown to be in the right positions within the cochleae based on postoperative temporal bone CT scans (Fig. 4).

## 4. Discussion

CI is recognized as a safe and secure approach for patients with normal ears as well as those with malformations of the inner structures.^[5,6]^ Yet, in some cases where multiple malformations such as microtia, inner ear malformations or even laryngomalacia are present, surgery can be extremely difficult. In this context, the current case report seeks to inform otologists on practices for preoperatively, and intraoperatively and postoperatively managing such patients.

Firstly, systematic preoperative preparation and evaluation are required. At this stage where a child with multiple anomalies is preoperatively screened, the most likely outcome of CI, including the best timing that will lead to the best outcome needs to be considered. At the same time, emphasis is laid on the preoperative value of CT/MRI scans. Indeed, CT imaging helps to clearly distinguish between structures such as mastoid pneumatization, facial nerve, middle ear, and cochlea and the bony labyrinth. Similarly, MRI offers the possibility of visualizing the vestibulocochlear nerve anatomy within the internal auditory canal, with this being an important requirement before proceeding for CI. In addition, patients with middle and inner ear malformations should be carefully assessed for venous malformations of the temporal bone. In particular, patients who are highly at risk of airway abnormalities need to be preoperatively identified in order to plan their perioperative airway management. This can be useful as administration of general anesthesia in the presence of laryngomalacia carries certain risk of mortality, especially in the case of exacerbation of airway reactivity and apnea in the postoperative period, as prolonged intubation including mechanical ventilation may then be required.^[7]^ For such patients, the medical history need to be carefully assessed in view of identifying positions and/or situations which are conducive to worsening or improving obstructive symptoms. At the same time, strategies such as lateral recumbent positioning, administration of supplemental oxygen and being prepared for reintubation in order to prevent extubation failure need to be in place. Due to the risk of total airway obstruction, it can be challenging to manage these patients, and as such, a well thought-out plan for airway management, including readiness to potentially perform a tracheotomy should be discussed during preoperative counseling.

Secondly, it is important to prepare multiple methods of identifying facial nerve and Scala tympani of the cochlea. Standard CI is carried out on the posterior tympanum through a trans-mastoid facial recess approach that opens the facial nerve recess to expose the round window niche and round window membrane. However, according to Hoffman et al^[8]^, up to 16% of patients with cochlear hypoplasia may exhibit an abnormal anatomy of the facial nerve, with the frequency and severity of this nerve anomaly being correlated with the severity of the microtia.^[9]^ Anomalies in the intratympanic course of the facial nerve have been associated with inner ear malformation, while in microtia, an abnormally placed mastoid course of facial nerve is more likely to be encountered. This emphasizes the benefit of intraoperative facial nerve monitoring as well as the need to have high requirements for surgeons about adequate psychological preparation and good microsurgical foundation to deal with vascular variation and nerve anomaly. In the current case, restricted trans-mastoid access to the facial recess was more likely to be observed in the presence of a poorly developed mastoid cavity combined with a low-lying tegmen. However, there were almost no anatomical landmarks in the mastoid cavity for reference during the operation, especially vascular malformation and significantly altered anatomical structures of the facial recess, which could have led to the failure of operation or even serious complications such as injury to the facial nerve. The position of the facial nerve in relation to the stapes was significant and allowed the nerve to be located and preserved. When dealing with cochlear hypoplasia, the surgeon must be ready with multiple techniques and special electrodes that will assist implantation. Cochlear hypoplasia leads to shorter cochlea and may require alternative electrode arrays or insertion techniques. It is not always possible to use the standard electrode arrays in the setting of the cochlear hypoplasia. In our case, a short electrode FLEX24 (19 contacts spread over 20.9 mm active length) was fully inserted without difficulty.

## 5. Conclusion

This case report of CI presents a patient with profound sensorineural hearing loss, along with laryngomalacia, vascular anomalies and severe middle and inner ear malformation. With the procedure being challenging, the main issues encountered preoperatively, intraoperatively and postoperatively as a result of the middle and inner ear malformation are discussed.

## Author contributions

**Conceptualization:** Xue Gao, Pu Dai.

**Data curation:** Juan Zhao, Xi Wang, Xing Liu, Min Liu, Meng-Meng Guo.

**Formal analysis:** Zhen-Dong Wang, Ya-Yan Lu, Jia Li, Yong Feng, Kun Yang.

**Investigation:** Xue Gao, Pu Dai.

**Resources:** Jin-Cao Xu.

**Supervision:** Xue Gao, Jin-Cao Xu, Pu Dai.

**Validation:** Xue Gao, Guan-Hua Li, Xi Wang, Wei Wang.

**Writing – original draft:** Xue Gao, Juan Zhao.

**Writing – review & editing:** Pu Dai.
